# Gender (r)evolution and contemporary psychiatry

**DOI:** 10.1192/bjo.2022.46

**Published:** 2022-04-07

**Authors:** Yulia Furlong, Aleksandar Janca

**Affiliations:** Dr Yulia Furlong is a Senior Clinical Lecturer at the University of Western Australia, and Consultant Psychiatrist and the Head of Service for CAMHS Crisis Connect at Perth Children's Hospital. Dr Furlong was the Head of Service for the Paediatric Consultation Liaison and Gender Diversity Service at Perth Children's Hospital until January 2022; Prof. Aleksandar Janca is Emeritus Professor of Psychiatry and Director of the WHO Collaborating Centre at the University of Western Australia.

**Keywords:** Sexual and gender identity disorders, nosology, phenomenology, childhood experience, classification

## Abstract

Perlson et al's editorial ‘Envisioning a future for transgender and gender-diverse people beyond the DSM’ heralds the arrival of the ICD-11's gender incongruence categories among conditions related to sexual health, brightening the spotlight on the re- (or rather de-)classification of gender-related disorders, which is a step in the right direction.

Perlson et al's recent editorial ‘Envisioning a future for transgender and gender-diverse people beyond the DSM’^1^ heralds the arrival of the ICD-11's gender incongruence diagnostic categories, brightening the spotlight on the re- (or rather de-) classification of gender disorders. The 21st century has witnessed the paradigmatic shift of gender identities existing beyond the binary. Perlson et al provide good reasoning on why, in the absence of mental disorder, some transgender and gender-diverse (TGD) individuals still need circumscribed psychiatric services, but one is always mindful of throwing the metaphorical baby out with the bathwater, and the ‘wholesale removal of gender dysphoria from DSM-5’ is a notion that piques curiosity – perhaps something to be considered in the next revisions of the DSM.

Gender-related diagnoses are still relatively new to the contemporary diagnostic classifications, with ‘transsexualism’ appearing for the first time in 1975 in the ICD-9, under the category of ‘sexual deviations’, alongside ‘transvestism’.^2^ The progressive work of Harry Benjamin, who is credited as the founding father of Western transsexualism, helped to distinguish between transvestism and transsexualism,^3,4^ consequently sending this taxonomy into an evolutionary loop that resulted in the current thinking behind the new ICD-11's gender incongruence diagnoses. See Table 1 for an overview of shifting diagnostic terms that have been utilised in ICD and DSM diagnostic classifications over the years.

**Table 1:** Taxonomy in evolution: situating gender-related diagnoses in the ICD and DSM

Classification manual and edition	Gender-related taxonomy	Category
ICD-9 (1975)	Transvestism and transsexualism	Sexual deviations
DSM-III (1980)	Transsexualism	Psychosexual disorders
DSM-III-R (1987)	Transsexualism	Disorders usually first evident in infancy, childhood or adolescence
ICD-10 (1990)	Transsexualism	Gender identity disorders
DSM-IV (1994) and DSM-IV-TR (2000)	Gender identity disorder in adolescents or adults	Sexual and gender identity disorders
DSM-5 (2015)	Gender dysphoria in adolescents or adults	Gender dysphoria
ICD-11 (2021)	Gender incongruence of adolescents and adults; Gender incongruence of childhood	Conditions related to sexual health (moved out of Chapter 5 ‘Mental disorders’)

The ICD-11 is ready for worldwide implementation in 2022, with all diagnostic categories and specifiers already published for external consultation.^5^ To avoid the stigma of being diagnosed with a mental disorder and acknowledging that classifying gender identity in this way can cause enormous stigma for people who are transgender, the ICD-11 has redefined and reclassified TGD presentations by placing them in Chapter 17 ‘Conditions related to sexual health’, which is a decisive step in the right direction. Compared with the DSM-5's classification of gender dysphoria, neither mental distress nor mental dysfunction is required to fulfil the new ICD-11 constructs of gender incongruence, which have replaced ‘state of intense distress’ with ‘feelings of dislike or discomfort of sexual anatomy or anticipated secondary sex characteristics’, in addition to emphasising that gender-variant behaviour and preferences alone are not sufficient for a diagnosis. When it comes to TGD individuals, the ICD-11 shifts focus away from psychopathology and a deficit-based assessment, toward acknowledgement of the intrinsic aspects of gender incongruence.^6^ There is growing evidence in favour of these proposed modifications;^7^ a recent multicentre, international study of 649 transgender adults found empirical evidence of the greater specificity and discriminability of the ICD-11 when compared with DSM-5 diagnostic characteristics. Methodologically, this study used receiver operating characteristic and item response theory to analyse a latent construct that is represented by the value of each diagnostic requirement, including four main manifestations of ‘gender incongruence’/‘gender dysphoria’ that are shared in both systems, as well as a duration requirement and the presence of mental distress/dysfunction related only to the DSM-5 classification of gender dysphoria.^6^

ICD-11 Chapter 24 Q codes (‘Factors that influence health status or contact with health services’), including QA1Y (‘Contact with health services for other specified counselling’) and QA34 (‘Contact with health services for fertility preservation counselling’) further highlight the refinement of new classification toward recognising specific health needs of TGD individuals and making a case for dedicated services that scaffold social and medical transition. The ICD-11 category of HA60 ‘Gender incongruence of adolescents and adults’ reduces symptom duration to several months, theoretically expediting vital service access for this cohort with presumed diagnostic stability. In contrast, the ICD-11 category of HA61 ‘Gender incongruence of childhood’ necessitates a 2-year timeframe for diagnosis, a reflective inclusion of a criterion that acknowledges the individual and evanescent journey on which these young people (and their families) embark, as well as the complexities and flux that an emerging (rather than stable) diagnostic construct encounters.^8^ It remains to be seen if this classification redesign will be empirically validated. It is envisaged that the inclusion of the ‘Gender incongruence of childhood’ category in the ICD-11 will provide better recognition and acceptance of earlier transgender experience and create the framework for developing age-appropriate clinical care, as well as renewing effort to assist clinicians, young people and family members in navigating the healthcare pathways, and helping to address the medico-legal challenges and other obstacles many transgender and non-binary children encounter when attempting to access gender-affirming care. With regard to non-binary presentations, it should be noted that neither the ICD-11 nor the DSM-5 diagnostic classifications specifically address issues relating to experiences of persons identifying as agender, androgyne, bigender, demigender and ‘other gender identities’ (as they are called in DSM), which can be seen as a step forward in depathologising such non-binary orientations in the current revisions of our diagnostic classifications.^9^ Any potential inclusion of such gender orientations in the future would require development of more sensitive clinical instruments and diagnostic criteria that reflect the non-binary gender experience.

The World Health Organization's reclassification of conditions that are related to sexuality and gender identity demonstrates the progress the organisation has made over the past years in its fight of the stigma and human rights violations that exist in the intersection of transgender status and mental illness. A far cry from 2017, when a trailblazing Denmark, without the World Health Organization's support, refused the utilisation of psychiatric diagnostic categories in relation to TGD citizens in their efforts to depathologise gender diversity.^10^ Despite these changes, the psychiatrist may still be a necessary participant in the treatment journey of TGD individuals, although our diagnostic labels for this cohort may be reduced to stress-related and adjustment categories. We believe that, theoretically, the ICD-11 has found a sound solution to the complex task of keeping a balance between concerns relating to the unnecessary stigmatisation of TGD people and the need for diagnostic categories that support the provision of the dedicated healthcare services they may require. We will watch closely at what comes next, and evaluate how well this new diagnostic model performs in practice.
